# The study of direct and indirect effects of radiofrequency ablation on tumor microenvironment in liver tumor animal model

**DOI:** 10.1186/s12885-022-09730-x

**Published:** 2022-06-17

**Authors:** An-Na Jiang, Bing Wang, Song Wang, Kun Zhao, Hao Wu, Kun Yan, Wei Wu, Wei Yang

**Affiliations:** Department of Ultrasound, Peking University Cancer Hospital & Institute, Key laboratory of Carcinogenesis and Translational Research (Ministry of Education/Beijing), Beijing, 100142 China

**Keywords:** Radiofrequency ablation, Liver tumor, Tumor microenvironment, Direct effect, Indirect effect

## Abstract

**Background:**

Direct and indirect effects of radiofrequency ablation (RFA) on tumor microenvironment of the liver tumor have been noted, which was reported to be related to a variety of tyrosine protein kinase or cytokinetic pathway, but have not been thoroughly investigated and conclusive.

**Purpose:**

To elucidate direct and indirect effects of RFA on tumor microenvironment in the liver tumor model, and to explore the role of the specific inhibitor in tumor growth by targeting the key pathway of RFA.

**Materials and methods:**

One hundred and ten mice with H22 liver tumor were used in animal experiments. Eighty-four mice were randomized into three groups: control, direct RFA and indirect RFA (a block slide was inside the middle of the tumor). The growth rate of the residual tumor after RFA was calculated (*n* = 8 each group) and the pathologic changes at different time points (6 h, 24 h, 72 h and 7d after RFA) were evaluated (*n* = 5 in each subgroup). After semi-quantitative analysis of the pathological staining, the most significant marker after RFA was selected. Then, the specific inhibitor (PHA) was applied with RFA and the tumor growth and pathological changes were evaluated and compared with RFA alone. The Kruskal-Wallis test was used for evaluating the significance of different treatments in the pathological positive rate of specific markers in tumor. The two-way analysis of variance was used to determine the significance of treatment in tumor growth or body weight.

**Results:**

The growth rate of the residual tumor in the direct RFA group was faster than the indirect RFA group (*P* = 0.026). The pathological analysis showed the expression of HSP70 (73 ± 13% vs 27 ± 9% at 24 h, *P* < 0.001), SMA (70 ± 18% vs 18 ± 7% at 6 h, *P* < 0.001) and Ki-67 (51 ± 11% vs 33 ± 14% at 7d, *P* < 0.001) in the direct RFA group was higher than those in the indirect RFA group after RFA. On the other hand, the expression of c-Met (38 ± 11% vs 28 ± 9% at 24 h, *P* = 0.01), IL-6 (41 ± 10% vs 25 ± 9% at 24 h, *P* < 0.001) and HIF-α (48 ± 10% vs 28 ± 8% at 24 h, *P* < 0.001) in the indirect RFA group was higher than those in the direct RFA group. And the expression of c-Met increased mostly in both direct and indirect RFA group compared to the baseline (53 and 65% at 72 h). Then the specific inhibitor of c-Met-PHA was applied with RFA. The growth rate of the tumor was significantly slower in the RFA + PHA group than the RFA alone group (1112.9 ± 465.6 mm^3^ vs 2162.7 ± 911.1 mm^3^ at day 16, *P* = 0.02).

**Conclusion:**

Direct and indirect effects of RFA on tumor microenvironment changed at different time points and resulted in increased residual tumor growth in the animal model. It can be potentially neutralized with specific inhibitor of related pathways, such as tyrosine-protein kinase c-Met.

**Supplementary Information:**

The online version contains supplementary material available at 10.1186/s12885-022-09730-x.

## Background

Radiofrequency ablation (RFA) is a minimally invasive treatment used in a wide range of tumors including primary solid tumors and tumor metastasis. Because of its advantages of safety, easy operation, effective and low cost, RFA is now a common clinical therapy for many tumors such as hepatocellular carcinoma (HCC). RFA, known as a kind of thermal ablation, eliminate the tumor in situ through frictional heat and heat conduction produced by the ionic agitation to achieve thermal coagulative necrosis [1–3]. During ablation, the thermal energy does not only cause hyperthermic injury to the tumor cells, but also affect the tumor microenvironment [4]. After ablation, the electrode tip is surrounded by three zones. The central zone is completely necrosis, the peripheral zone is of sublethal hyperthermia and can be recovered from reversible injury, while the surrounding tissue is not affected by the thermal injury directly [5]. The process of tumor microenvironment changes occurs in at least two ways, through direct and indirect heat mechanisms [6].

It was reported that inflammatory infiltrates occurred in the peripheral zone, which was adjacent to the central coagulation zone due to the heat effect [7–9]. And the tumor cells, as well as the destroyed extracellular matrix and tissue components could release pro-inflammatory cytokines and trigger the release of additional cytokines, chemokines and vascular adhesion molecules [10–13]. So that the normal surrounding tissue could also be affected even though it was not directly heated. Some studies have identified several molecular mechanistic pathways that may contribute to the tumor microenvironment changes after RFA, such as IL-6, HGF/c-Met and VEGF, which were considered relevant to tumor growth, metastasis, and invasion [14, 15]. However, the direct and indirect effects of RFA on the tumor have been noted but not thoroughly investigated. Accordingly, the purpose of our study was to explore the potential mechanism of the heat effects after ablation and evaluate the key cytokinetic to improve the treatment outcome.

## Materials and methods

### Experimental overview

The study was approved by Institutional Animal Care and Use Committee (Peking University, Cancer Hospital) before the start. And H22 hepatoma (ATCC, Manassas, Va) in BALB/C mice (Vital River Experimental Animal Technology, Beijing, China) were used in this study. This research was performed in three phases to systematically investigate the potential heat effects (direct and indirect effects) of RFA on liver tumor and the related adjuvant therapy.

### Study design

**Fig. 1:**
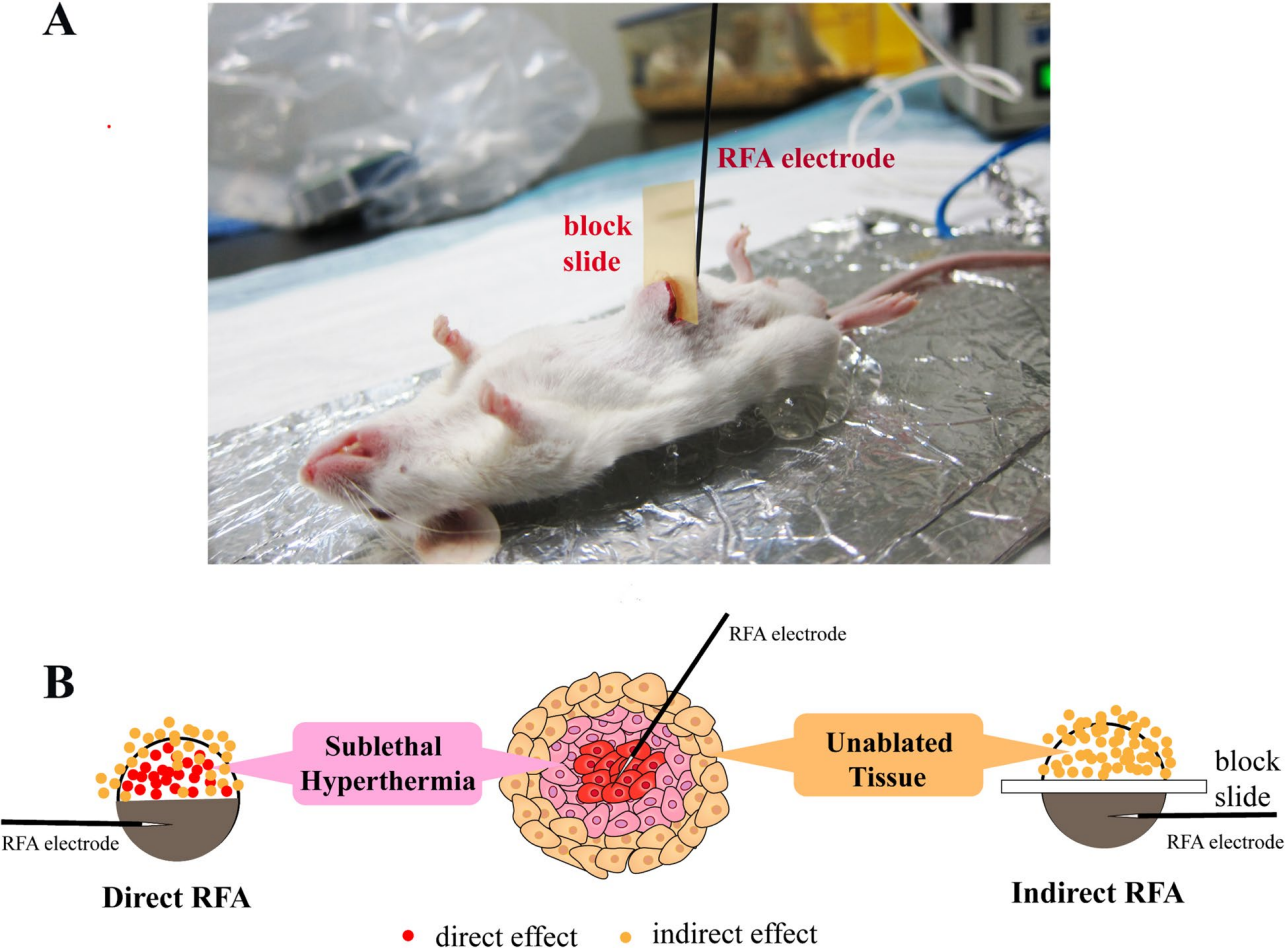
Illustration of direct and indirect RFA of liver tumor in the animal model. **A** Photo of a tumor bearing mouse in the indirect RFA group. **B** Sketch diagram of direct and indirect effects of RFA on liver tumor. The grey region indicates the tumor necrosis. Red dots and yellow dots indicate the direct and indirect effects of RFA respectively

We performed our experiment by using a well-developed H22 tumor bearing animal model. With the tumor model, we divided the tumor into two parts in the middle with or without a thin block slide set inside. We designed two manners to conduct RFA which would mimic the effect of direct RFA or indirect RFA on tumor microenvironment. In the first manner, the RFA electrode was inserted into one half of the tumor. The ablation was conducted without the block slide between the two halves of the tumor. Thus, the other half of tumor was affected by the direct effect of RFA. In the second manner, the RFA electrode was inserted into one half of tumor. The ablation was conducted with the block slide between the two halves of the tumor. The slide blocked the heat transmission to the other half of the tumor. Thus, the other half of tumor was affected by the indirect effect of RFA. The unablated part of the tumors after RFA in the two manners were evaluated in the following days (Fig. 1).

### Comparison of tumor growth rates

Twenty-four mice with H22 tumors were used in this phase, and the effect of the two different manners on the growth of the unablated half of the tumor after RFA was analyzed. On the basis of our previous work [16, 17], the average diameter of tumor destruction after RFA was around 7 mm, while the risk of spontaneous necrosis was higher when the tumor was larger than 15 mm. Accordingly, the range of 7–15 mm diameter was selected as an appropriate tumor size for RFA. In this study, the range of 10-14 mm diameter was selected as the appropriate tumor size when we performed RFA treatment. The mice were randomized into the following three groups (*n* = 8 in each group): (a) control, (b) direct RFA (without the block slide); (c) indirect RFA (with the block slide). The diameter of the residual tumor after RFA of each mouse was measured with caliper every 2 days. The tumor growth curves were calculated and compared among different groups.

## Assessment of pathologic findings

A total of sixty mice with H22 tumors were used in this phase and were randomized into the three groups: (a) control, (b) direct RFA, (c) indirect RFA. The mice were sacrificed at different time points after RFA, including 6 h, 24 h,72 h and 7 days for pathological analysis (*n* = 5 for each subgroup). Tissue was fixed in 10% formalin overnight at 4 °C, embedded in paraffin, and sliced at a thickness of 5 μm. The tissue was stained with hematoxylin eosin for gross pathologic examination. The specific immunohistochemistry (IHC) or immunofluorescence (IF) staining was used to detect the changes of the tumor microenvironment, containing the expression of the following markers, HSP70 (ab181606, Abcam, Cambridge, MA), SMA (ab32575, Abcam, Cambridge, MA), Ki-67 (12202S, Cell Signaling, Danvers, MA), VEGF (AF469, R&D Systems), CD31 (77,699 T, Cell Signaling, Danvers, MA), HIF-α (MAB1536-SP, R&D Systems), IL-6 (AF-406-SP, R&D Systems) and c-Met (AF527, R&D Systems).

The positive cells of different markers were analyzed according to IHC/IF staining of tumor specimen at 6 h, 24 h, 72 h and 7d after RFA. The expression and distribution of the markers in each specimen were observed under the microscope, and were analyzed blindly to the treatment to eliminate bias. The positive rate of each marker was calculated for five random high-power fields in each slide. The expression of each marker in different time points was semi quantitatively presented by bar graph. We analyzed the quantitative data of pathological results and evaluated the possible target markers that may have the most significant changes after RFA.

### Evaluation of the effect of specific inhibitor

In the third phase, we evaluated the role of inhibiting one of the key pathways underlying post ablation tumor response. The effect of an adjuvant small-molecule c-Met receptor inhibitor (PHA-665752, subsequently referred to as PHA; Sigma-Aldrich, St Louis, Mo) on tumor microenvironment after RFA ablation was studied. Sixteen mice with H22 tumors were used for tumor growth study. In this phase, the range of 8-10 mm diameter was selected as the appropriate tumor size when we performed RFA treatment. The mice were randomized into two groups (*n* = 8 in each group): (a) RFA alone, (b) PHA + RFA. PHA (0.83 mg/kg, 200 μl each) was injected intraperitoneally every 2 days for four times. RFA was performed 2 h after first injection of PHA. To mimic the residual tumor during ablation of large tumors in clinical practice, about half of the tumor was completely ablated. The diameter of the tumor and the body weight of each mouse was measured every 2 days. Next, 10 mice from the above two groups were sacrificed at 7 days after RFA (24 h after the last injection of PHA) (*n* = 5 in each group) for pathological analysis. IHC staining was used to compare the changes of the tumor microenvironment after administration of PHA, including Ki-67, CD31, HIF-α and c-Met.

### Animal model

For all experiments and procedures, anesthesia was induced by means of intraperitoneal injection of pentobarbital sodium (45 mg/kg, chemical reagent factory of Foshan, China). Animals were sacrificed in a CO_2_ chamber. RFA was performed in an established H22 liver adenocarcinoma model in BALB/C mice (female, weighing 18–20 g, aged 6–8 weeks). 0.2 ml of H22 cells (at a density of 1 × 10^7^/ml) suspended in serum-free RPMI-1640 and matrigel (1:1) were injected subcutaneously into the abdominal wall with an 18-gauge needle for each tumor to establish the liver adenocarcinoma model. Animals were monitored every 2 or 3 days to measure tumor growth. Ultrasonography was used to determine solid nonnecrotic tumors before treatment in this study. Mean starting tumor size was similar for all comparative treatment groups at initial treatment. The longitudinal and transverse directions of the tumor were measured with mechanical calipers and the body weight of the mouse was weighed on the electronic balance every 2 days after RFA. The measurement was performed by A-NJ and KZ, with 4 and 3 years of experience in animal experiments, respectively and verified by WY, with 12 years of experience in animal experiments, who was blinded to the treatment group. Tumor volume was calculated as D*d^2^*0.5, where D and d were the two diameters of the tumor measured above.

### RFA procedure

In the animal experiments, the 17-gauge monopolar electrode (ACT1507 electrode; Valleylab, Tyco Healthcare) and the 480-kHz RFA generator (Model CC-1-220; Valleylab, Tyco Healthcare, USA) were used during RFA. The animal was shaved off on the back and applied electrolytic contact gel and then placed on the conventional metallic grounding pad (Cosman Medical, Inc. USA) to complete the RFA circuit. About 0.7 cm of the electrode tip was placed at the center of the target tumor part first and the RFA generator was set to the tip temperature at 65 ± 2 °C and applied for 5 min. Here, we chose temperature 65 °C as the ablation parameter because the temperature point was the highest level for mice can tolerate during RFA treatment.

### IHC/IF staining

The paraffin tissue sections were firstly soaked in xylene to deplete paraffin, rinsed in ethanol to deplete xylene and soaked in hydrogen peroxide solution to reduce endogenous peroxidase activity. Then, antigen retrieval was performed by placing slides in a container and heated (97 °C) to enhance the accessibility of antibody to antigen for 10 minutes. After the slides were rinsed with PBS, they were blocked in goat serum to reduce nonspecific binding of the antibody. The blocked tissue slides were incubated with primary antibodies overnight. After washed with PBS, they were incubated with species matched secondary antibodies for 20 minutes and washed. For IHC reactions, the slides were then incubated with DAB (Beyotime Institute of Biotechnology) until desired stain intensity was observed, counterstained with hematoxylin and rinsed. Then, the slides were dehydrated in ethanol and emerged in xylene. After that, the slides were covered with cover glass and dried on a flat surface. For IF reactions, the slides were then incubated with DAPI (Beyotime Institute of Biotechnology) to visualize nucleic acid in each cell. After washed with PBS, anti-fluorescence quenching agent (Beyotime Institute of Biotechnology) was added. Then the slides were covered with cover glass, dried and kept in the dark to prevent bleaching fluorescent signal. Pathological slides were imaged and analyzed by using a microscope (Micromaster I; Westover Scientific, Mill Creek, Wash). For quantitative analysis, the percent of positively stained cells was counted on at least five high power (× 400) fields per slide and was assigned scores in a blinded fashion to remove observer bias [18].

### Statistical analysis

In this study, SPSS 27.0 software (SPSS, Chicago, IL, USA) was used for statistical analysis. *P* < 0.05 was statistically significant. All continuous data were provided as means ± SD. Kruskal Wallis test was used to evaluate the significance of different treatments. When the total *P* was less than 0.05, Nemenyi test was used for multiple comparison. The two-way analysis of variance was used to determine the significance of treatment in tumor growth or body weight.

## Results

### Comparison of tumor growth after direct or indirect RFA

**Fig. 2:**
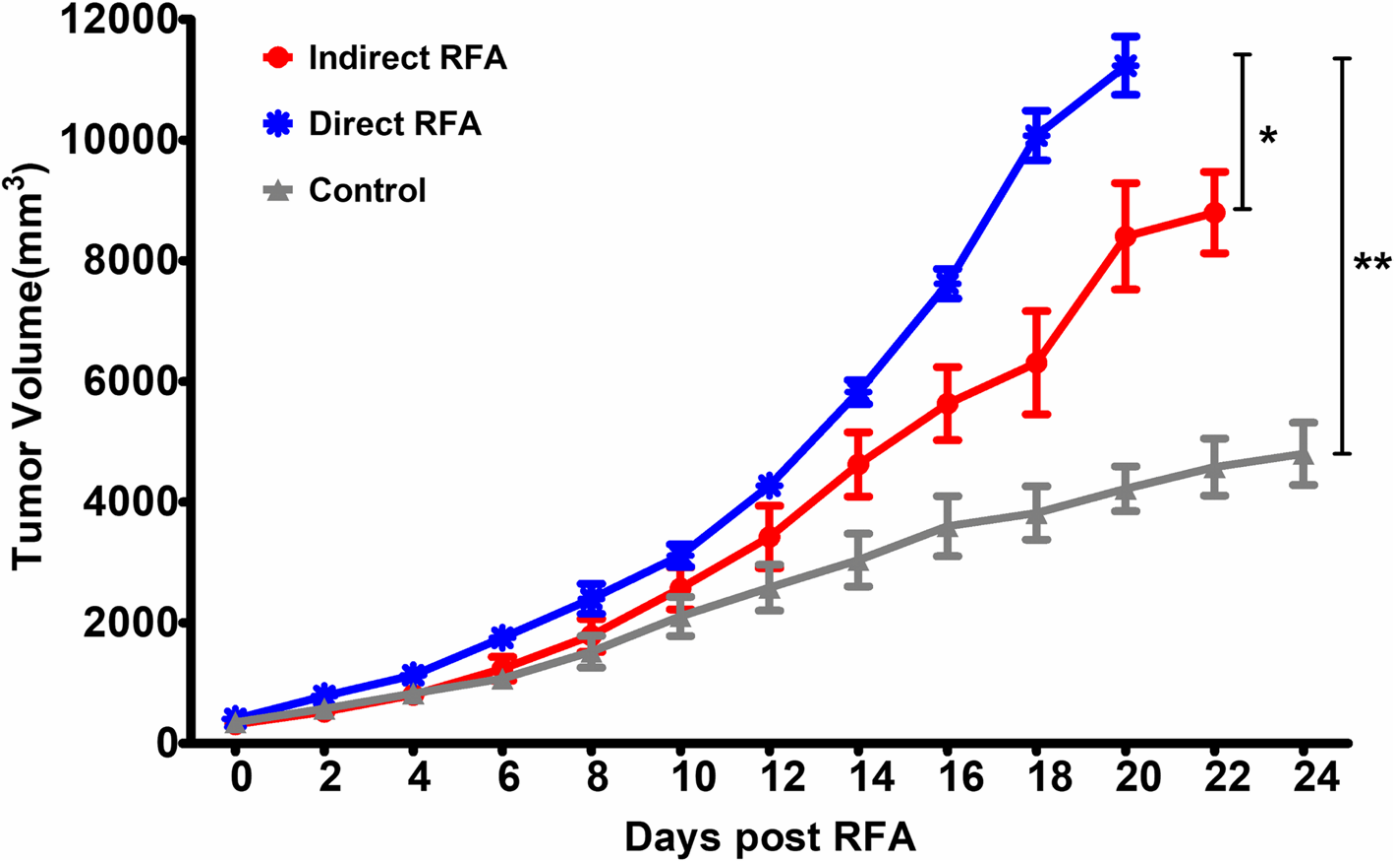
Comparison of tumor growth rate in mice bearing H22 liver tumor. The tumor growth curves showed the residual tumor grew faster in the direct RFA group than the indirect RFA group (*P* = 0.026). The tumor growth rate in the control group was the slowest compared with that in the direct RFA group (*P* < 0.001) and indirect RFA group (*P* = 0.042). *P < 0.05, **P < 0.01

The tumor growth curves showed the residual tumor grew faster in the direct RFA group than the indirect RFA group (*P* = 0.026). And the growth rate of residual tumor in the direct RFA group (*P* < 0.001) or in the indirect RFA group (*P* = 0.042) was faster compared with that in the control group (Fig. 2).

### Pathological changes after direct or indirect RFA

**Fig. 3:**
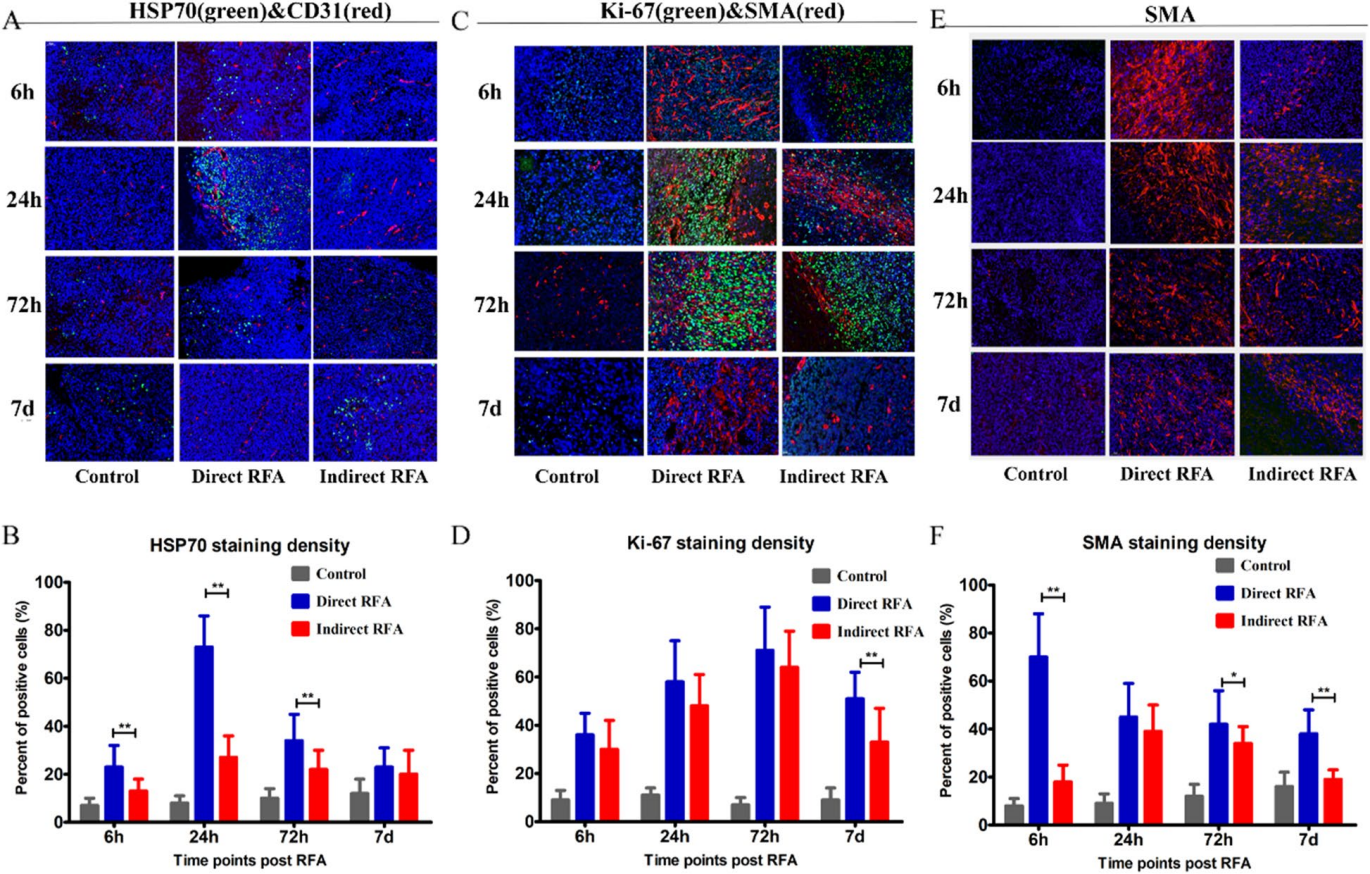
Assessment of pathologic findings obtained at 6 h, 24 h, 72 h and 7 days after treatment in different groups and the semi-quantitative analysis of HSP70 (**A-B**), Ki-67 (**C-D**) and SMA (**E-F**). The expression of HSP70 (green) was increased after RFA and reached the peak at 24 h in the direct RFA group, and was significantly higher than that in the indirect RFA group at 24 h. The expression of Ki-67 (green) increased after RFA and reached the peak at 72 h in both direct RFA and indirect RFA groups. The expression of SMA (red) was higher in the direct RFA group than the indirect RFA group after RFA and reached its peak at 6 h in the direct RFA group. The bar graph showed the percentage of positive cells among the direct RFA group (blue), indirect RFA group (red) and control group (grey) at different time points after RFA. *P < 0.05, **P < 0.01. The representative microphotographs were under 200 × magnification (**A**, **C**, **E**)

**Fig. 4:**
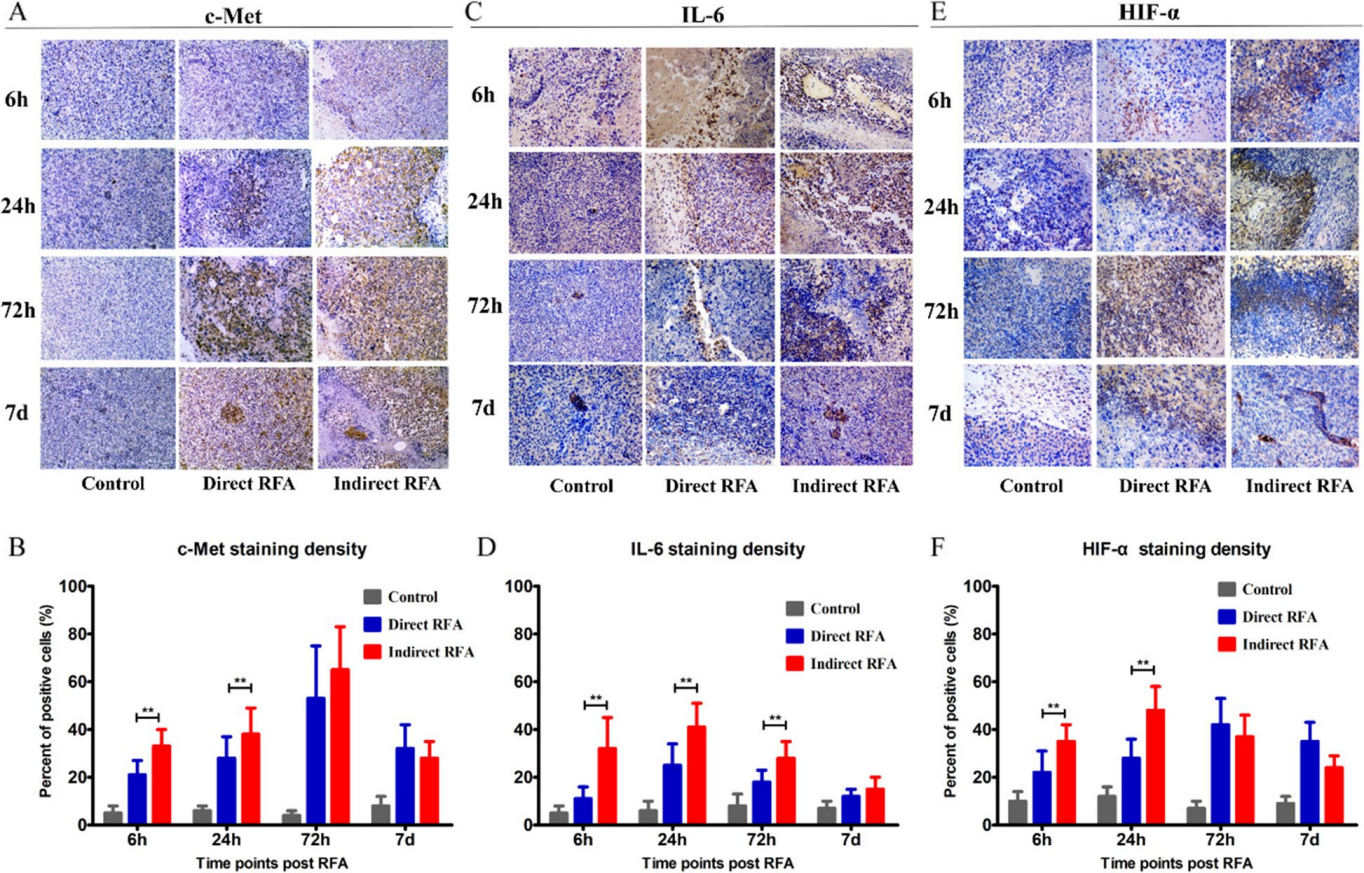
Assessment of pathologic findings obtained at 6 h, 24 h, 72 h and 7 days after treatment in different groups and the semi-quantitative analysis of c-Met (**A-B**), IL-6 (**C-D**) and HIF-α (**E-F**). The expression of c-Met increased after RFA and reached the peak at 72 h (> 50% positive rate) in both direct RFA and indirect RFA groups. The expression of IL-6 increased after RFA in the indirect RFA group and it was significantly higher than the direct RFA group from 6 h to 72 h after treatment. The expression of HIF-α was higher in the indirect RFA group than the direct RFA group from 6 h to 24 h after RFA. The bar graph showed the percentage of positive cells among the direct RFA group (blue), indirect RFA group (red) and control group (grey) at different time points after RFA. **P < 0.001. The representative microphotographs were under 200 × magnification (**A**, **C**, **E**)

**Fig. 5:**
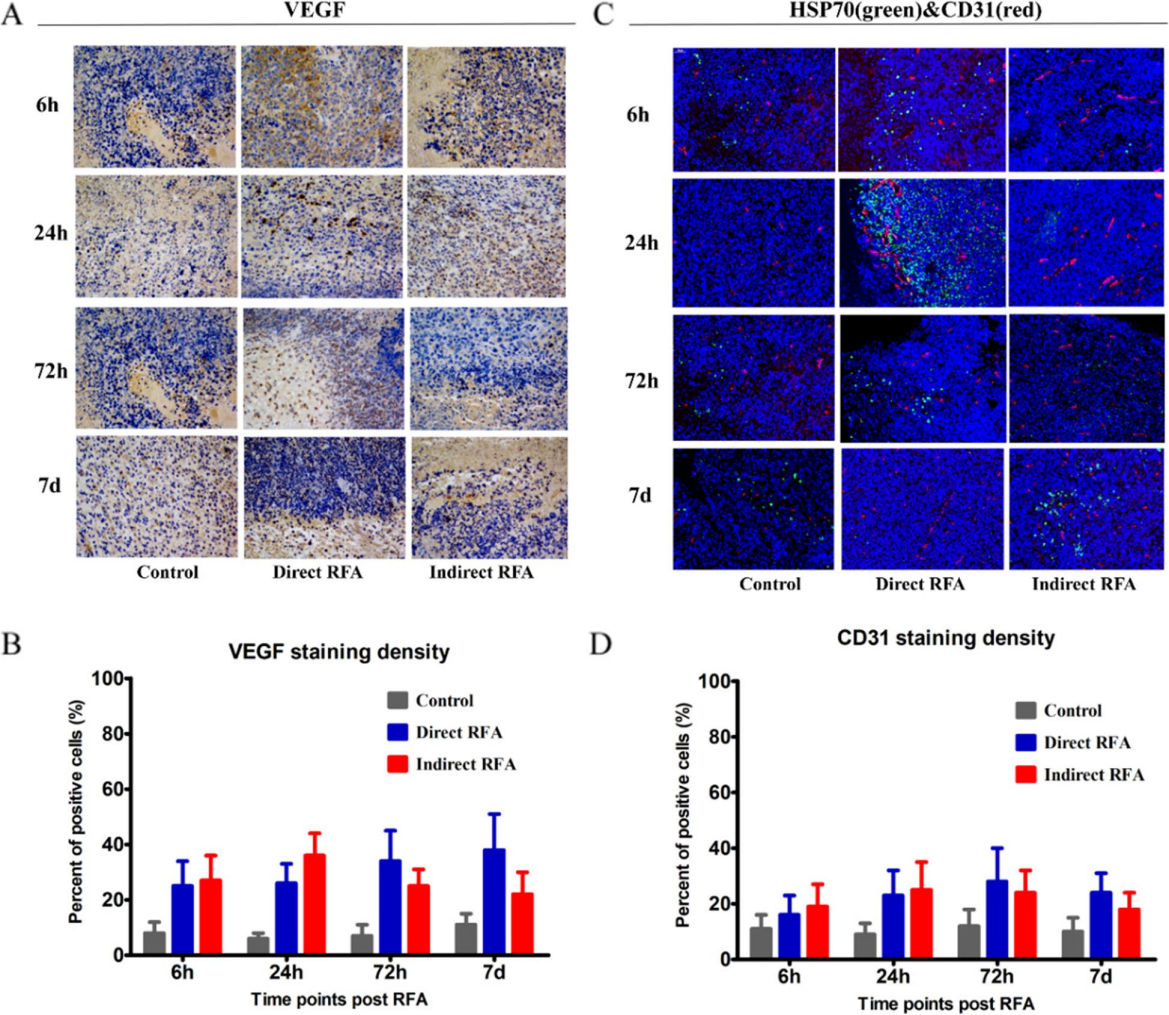
Assessment of pathologic findings obtained at 6 h, 24 h, 72 h and 7 days after treatment in different groups and the semi-quantitative analysis of VEGF (**A-B**) and CD31 (**C-D**). The expression of VEGF in the direct RFA group and indirect RFA group were always similar at all time points. The expression of CD31 (red) was increased at 24 h after RFA in both direct RFA and indirect RFA groups compared to the control group. The bar graph showed the percentage of positive cells among the direct RFA group (blue), indirect RFA group (red) and control group (grey) at different time points after RFA. The representative microphotographs were under 200 × magnification (**A**, **C**)

According to the IHC/IF staining of the tumor samples obtained at 6 h, 24 h, 72 h and 7d after RFA, we found significant changes with time manner in different groups on the pathological findings including HSP70, CD31, SMA, Ki-67, c-Met, IL-6, VEGF, and HIF-α (Figs. 3, 4 and 5).

After quantitative analysis, the dynamics changes of the different markers after direct RFA and indirect RFA were shown as follows. Firstly, the higher expression of the markers was found in the direct RFA group than the indirect RFA group in HSP70, SMA and Ki-67 markers. The expression of HSP70 reached the peak at 24 h after RFA in the direct RFA group and was significantly higher than that in the indirect RFA group (73 ± 13% vs 27 ± 9%, *P* < 0.001) (Fig. 3A-B). The expression of Ki-67 in the direct RFA group was increased greatly after treatment and it was significantly higher than the indirect RFA group (51 ± 11% vs 33 ± 14% at 7d, *P* < 0.001) (Fig. 3C-D). The expression of SMA reached the peak at 6 h after RFA in the direct RFA group and was significantly higher than that in the indirect RFA group (70 ± 18% vs 18 ± 7%, *P* < 0.001) (Fig. 3E-F).

Secondly, the higher expression of the c-Met, IL-6 markers was found in the indirect RFA group than the direct RFA group. The expression of c-Met in the indirect RFA group was higher than those in the direct RFA group from 6 h to 24 h after treatment (38 ± 11% vs 28 ± 9% at 24 h, *P* = 0.01), but there was no significant difference at 7 days after RFA (Fig. 4A-B). The expression of IL-6 in the indirect RFA group was higher than those in the direct RFA group (41 ± 10% vs 25 ± 9% at 24 h, *P* < 0.001) (Fig. 4C-D). Also, the expression of HIF-α in the indirect RFA group was higher than that in the direct RFA group (48 ± 10% vs 28 ± 8% at 24 h., *P* < 0.001) (Fig. 4E-F). On the other hand, the expression of VEGF (Fig. 5A-B) and CD31(Fig. 5C-D) in the direct RFA group and indirect RFA group were always similar at all time points.

Most importantly, the expression of c-Met (Fig. 4-B) and Ki-67 (Fig. 3-D) increased over 50% in both direct and indirect RFA group compared to the baseline (c-Met: 53% and 65% at 72 h after RFA; Ki-67: 71% and 64% at 72 h after RFA). Considering the possibility and feasibility of clinical application, c-Met was chosen as the target for inhibiting the key pathway in both direct and indirect effects of RFA.

### Effect of c-Met inhibitor in the tumor growth after RFA

**Fig. 6:**
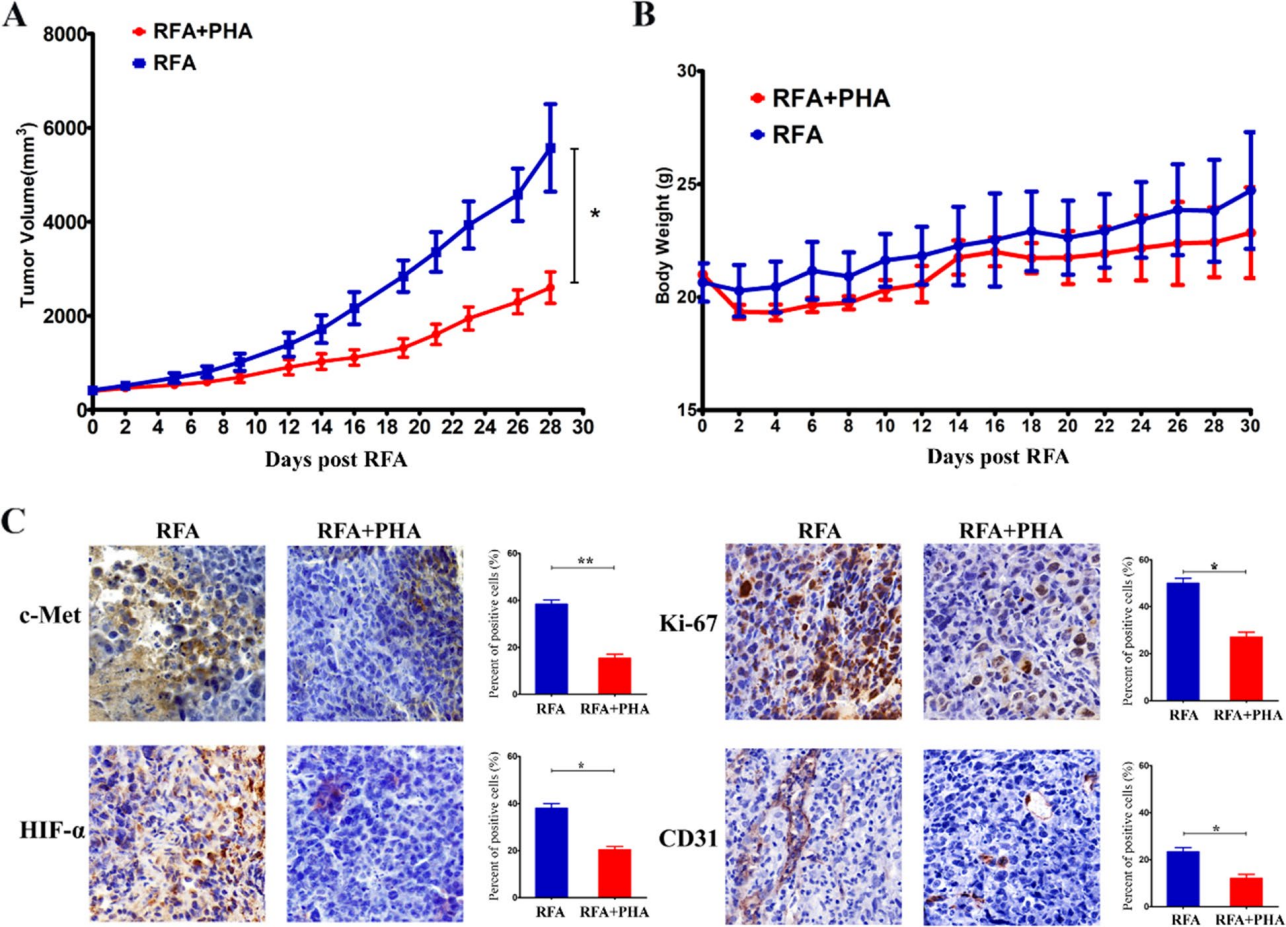
Effect of c-Met inhibitor in the tumor after RFA. **A** The tumor grew slower in the RFA + PHA group than the RFA group (*P* = 0.02). * *P* < 0.05. **B** During the period of follow-up, there were no obvious difference in the bodyweight change after treatment in the two experimental groups (*P* = 0.09). **C** Assessment of c-Met, HIF-α, Ki-67 and CD31 (brown) staining on pathologic findings obtained at 7 days after RFA in different groups. The expression decreased in the RFA + PHA group compared to the RFA group. **P < 0.001. The representative microphotographs were under 200 × magnification (C)

For H22 xenograft tumors, the measurements of the subcutaneous tumors in the two experimental groups were similar (RFA + PHA, 482.8 ± 169.8 mm^3^ vs RFA, 528.7 ± 78.4 mm^3^, *P* = 0.73) pretreatment. The tumor growth curve showed the tumor grew slower in the RFA + PHA group than the RFA group (*P* = 0.02) (Fig. 6-A). The difference began to be significant at 16 days after RFA (1112.9 ± 465.6 mm^3^ vs 2162.7 ± 911.1 mm^3^, *P* = 0.02). During the period of follow-up, there were no obvious changes in the health-related parameters after different treatment including: body weight (*P* = 0.09, Fig. 6-B), respiratory status, eating and drinking behaviors, response to stimulations, and general activity level.

IHC staining revealed that the expression of c-Met (38 ± 3% vs 15 ± 3%), HIF-α (38 ± 4% vs 20 ± 3%), Ki-67 (50 ± 4% vs 27 ± 4%) and CD31 (23 ± 3% vs 12 ± 3%) in the tumor were decreased at 7 days in the RFA + PHA group compared to the RFA group (*P* < 0.001 for all comparisons) (Fig. 6-C).

## Discussion

RFA is a minimally invasive treatment for tumors which has been confirmed satisfactory efficacy and low complication [19, 20]. During ablation, local hyperthermia caused by the electrode leads to the cell coagulation necrosis and the changes of the tumor microenvironment. Some studies have found that incomplete RFA could stimulate tumor invasiveness, manifested as accelerated growth and enhanced metastasis [21–23]. What’s more, it was reported that RFA induced not only a local periablational inflammatory zone but also the distant tumor growth [24–26]. Elevated serum levels of inflammatory cytokines, chemokines and growth factor might be an early indicator of the incomplete RFA and subsequently a potential tumor relapse [24, 27]. In our previous studies, we also found that residual tumor progressed more rapidly and developed several methods to improve the efficacy of RFA, but the effects to the tumor microenvironment was not yet clear [28, 29]. Therefore, in this study, we aimed to explore the effect of RFA to tumor microenvironment and look for the key cytokine or pathway for target therapy.

In our study, we developed the animal model to elucidate the direct and indirect effects of RFA to the tumor microenvironment. The tumor was divided into two halves in the middle before RFA and a glass slide was set inside to block the thermal transmission to the other half of the tumor (indirect RFA group). The temperature of the electrode tip was set at 65 ± 2 °C, applying 5 minutes and the diameter of necrosis area could reach 7 mm, so that half of the tumor was complete necrotic. In this way, the unablated tumor was regulated by the indirect effect caused by heat. On the other hand, the tumor in the direct RFA group was regulated by both direct heat effect and indirect effect. The direct effect refers to the ablation, which was closely related to heat shock (HSP70), inflammatory reaction for thermal damage (IL-6). Indirect effect refers to re-regulation of tissue metabolic function and cell proliferation (HIF-α, SMA, Ki-67), angiogenesis (VEGF, CD31) or growth factor (HGF, c-Met), which occurs even after ablation. We try to look for the key pathway which played an important role in both direct and indirect effects so that to find the potential target point for pharmacologic suppression of these effects.

We investigated the expression of several heat related markers including HSP70、SMA、VEGF、CD31、Ki-67、HIF-α, IL-6, and c-Met. The effects of RFA to the tumor microenvironment could be linked to the activation of IL-6/HGF/c-Met pathway which produce downstream angiogenesis and VEGF production [30]. These factors also play important roles in promoting tumor growth and invasiveness, which was evaluated by Ki-67、SMA and CD31 in our study [31–33]. The expression of SMA and Ki-67 were up-regulated after RFA and increased more notably in the direct RFA group, indicating the direct heat effect contributes to the tumorigenesis. While the c-Met and IL-6 were more pronounced positive expressed in the indirect RFA group, providing the evidence of stimulation of aggressive tumor biology. Meanwhile, we noticed several other potential pathways linked to the RFA effects, including hypoxia, which is related to tumor invasiveness [34]. The expression of HIF-α which reflects the hypoxic microenvironment, increased more significantly in the indirect RFA group, suggesting the host of secondary reactions after RFA. Additionally, heat stress responses was also another stimulation after RFA and was assessed by heat shock protein. HSP70 plays an important role in alleviating cell damage, enhancing anti-apoptotic ability and immune regulation [35, 36]. The high expression of HSP70 was observed in the direct RFA group 24 h after RFA, which was directly related to the heat affect. Our results provided the evidence of several mechanistic pathways contributing to the direct and indirect effect of RFA. It is noteworthy that the indirect effect also occurred rapidly after RFA (within hours to days), it was quite necessary to identify and eliminate the residual tumor as soon as possible. And the adjuvant therapy is particularly necessary when tumor residue is suspected. Interestingly, the expression of c-Met and Ki-67 showed the maximum level of upregulation in both direct and indirect RFA group compared to the baseline. Ki-67 refers to the proliferation of the tumor and the combination of the chemotherapeutic drugs with RFA has been studied a lot. Therefore, as a next step, we focused on the upregulation of the HGF/c-Met pathway after RFA, which was known as driving tumor growth, metastatic invasion, and aggressive tumor biology [37, 38]. It was reported that HGF/c-Met could lead to initiate several downstream signaling pathways including PI3K/AKT, Ras/MAPK, JAK/STAT, which were involved in tumor progression [39]. And the c-Met pathway was interconnected with VEGF and VEGF receptor, which promoted angiogenesis and endothelial cell growth [40]. Therefore, c-Met is a promising target for tumor and several HGF/c-Met targeting inhibitors and antibodies have been developed.

Gaurav Kumar reported that c-Met inhibitor could block HGF/c-Met pathway which mediated distant subcutaneous tumor growth after RFA of normal liver [30]. In this study, we explored the role of c-Met inhibitor in the residual progression after ablation of tumor. We demonstrated that activated pathways contributing to the effects of RFA could be blocked by combining adjuvant drug against key receptor target. The PHA inhibitor has a strong selectivity for c-Met and can induce apoptosis and cell cycle arrest [41]. According to the IHC staining, the expression of c-Met remained elevated until 7 days after RFA. Therefore, the PHA was applied four times to cover this period. RFA combined with PHA showed slower tumor growth, while RFA alone group showed positive tumor growth. Because the tumor after RFA was relatively small in size, the differences of early growth rate in different groups was not obvious. At 14 days after treatment, differences between RFA and RFA + PHA groups began to become apparent. No organ metastasis was found at the end of the follow up. Then, we investigated the expression of the related markers after the combination therapy including c-Met, HIF-α, Ki-67 and CD31. The results indicated the PHA could down regulate the expression of c-Met and influence the cell proliferation and tumor angiogenesis. These findings supported the role of adjuvant inhibition of c-Met via PHA could suppress RFA induced tumor progression. During the toxicity experiment, the PHA showed no special biological toxicity. We preliminarily verified the efficacy of the PHA in combination with RFA treatment through the animal experiments.

There were several limitations in our study. First, we just preliminarily explored the expression of some heat or inflammatory related markers through pathological staining, but the effect of ablation was more than that. The changes of immune microenvironment and serum protein profiles after RFA have not been studied. Comparative proteome research and subsequent validation are needed to clarify the mechanism of ablation effect. Second, we only evaluated the proliferation and angiogenesis under the combination of RFA and PHA, the further effects such as tumor stemness, immune suppression of blocking the c-Met pathway on the tumor microenvironment needed to be explored in the next step. PHA is an effective c-Met inhibitor, but the clinically available c-Met pathway inhibitors and its level of inhibition after RFA remains to be explored. Third, we studied the effect of RFA with only one cell line and one subcutaneous model, further characterization in other tumor cell lines and the use of orthotopic model are necessary to characterize the effects of different tumors and microenvironments. Furthermore, we chose radiofrequency as the energy source to ablate the subsequent tumor in this study as the treatment method, but other local treatment such as microwave ablation and interventional therapy are used clinically now. Other studies could be developed to determine whether similar effects herein are present.

In conclusion, the direct and indirect effects of RFA on tumor microenvironment occur at different times and result in acceleration growth of residual tumor growth in the animal model. The inhibitor of PHA could help suppress this phenomenon and achieve better overall outcome with combination of RFA.

## Supplementary Information

**Additional file 1.**

## Data Availability

The datasets used and/or analysed during the current study are available from the corresponding author on reasonable request.
